# Oxygen-permeable microwell device maintains islet mass and integrity during shipping

**DOI:** 10.1530/EC-17-0349

**Published:** 2018-02-26

**Authors:** Darling M Rojas-Canales, Michaela Waibel, Aurelien Forget, Daniella Penko, Jodie Nitschke, Fran J Harding, Bahman Delalat, Anton Blencowe, Thomas Loudovaris, Shane T Grey, Helen E Thomas, Thomas W H Kay, Chris J Drogemuller, Nicolas H Voelcker, Patrick T Coates

**Affiliations:** 1 The Centre for Clinical and Experimental Transplantation (CCET) The Royal Adelaide HospitalAdelaide, South Australia, Australia; 2 Cooperative Research Centre for Cell Therapy Manufacturing (CRC-CTM)Adelaide, South Australia, Australia; 3 Department of MedicineFaculty of Health and Medical Sciences, University of Adelaide, South Australia, Australia; 4 St Vincent’s Institute of Medical ResearchFitzroy, Victoria, Australia; 5 The University of MelbourneDepartment of Medicine, St. Vincent’s Hospital, Fitzroy, Victoria, Australia; 6 Science and Engineering FacultyQueensland University of Technology, Brisbane, Queensland, Australia; 7 Future Industries InstituteUniversity of South Australia, Mawson Lakes, South Australia, Australia; 8 School of Pharmacy and Medical SciencesUniversity of South Australia, Adelaide, South Australia, Australia; 9 Transplantation Immunology GroupGarvan Institute of Medical Research, Darlinghurst, New South Wales, Australia; 10 Monash Institute of Pharmaceutical SciencesMonash University, Parkville, Victoria, Australia

**Keywords:** islet, transplantation, hypoxia, shipping, microwell

## Abstract

Islet transplantation is currently the only minimally invasive therapy available for patients with type 1 diabetes that can lead to insulin independence; however, it is limited to only a small number of patients. Although clinical procedures have improved in the isolation and culture of islets, a large number of islets are still lost in the pre-transplant period, limiting the success of this treatment. Moreover, current practice includes islets being prepared at specialized centers, which are sometimes remote to the transplant location. Thus, a critical point of intervention to maintain the quality and quantity of isolated islets is during transportation between isolation centers and the transplanting hospitals, during which 20–40% of functional islets can be lost. The current study investigated the use of an oxygen-permeable PDMS microwell device for long-distance transportation of isolated islets. We demonstrate that the microwell device protected islets from aggregation during transport, maintaining viability and average islet size during shipping.

## Introduction

Diabetes mellitus affects about 390 million people worldwide, with a steady increase in global incidence over the last decades (1, 2). Of these, an estimated 7–12% are diagnosed with type 1 diabetes (T1D) (3). T1D patients are required to measure blood glucose levels and inject insulin multiple times per day and often experience severe impairment of quality of life and potential long-term effects such as microvascular complications and organ damage (4, 5, 6, 7, 8). Currently, there is no prevention or cure for T1D, and the only minimally invasive therapy available that can lead to insulin independence is islet transplantation. Since the publication of an improved protocol for islet isolation and transplantation by the Edmonton group in 2000 (9, 10), over 750 islet transplants have been performed globally (11). However, the procedure is currently limited to a small group of T1D patients with brittle diabetes or hypoglycemia unawareness (unawareness of critically low blood glucose levels) (12). The major reason for the limited availability of this therapy is one of supply: the dearth of suitable donor pancreata and the loss of functional insulin-producing islet tissue throughout the isolation and transplantation process (13, 14).

The process of isolating islets from the pancreas of a deceased organ donor follows a complex protocol that is both labor and time-intensive. This process requires expensive isolation facilities equipped with clean-room facilities operating to Good Manufacturing Practice (GMP) standards and staffed with a team of experienced professionals able to perform the isolation (15, 16). Therefore, to ensure the quality of the islet isolation process and to reduce the total costs associated with islet transplantation, it is advantageous to have centralized isolation facilities from where isolated islets are shipped to different clinical transplantation centers. This hub and spoke model approach requires the transport of islet tissue, often across long distances, while maintaining the quality and quantity of islets. Clinical islet transplant networks such as in the United Kingdom, the Swiss-French Gragil consortium, the Nordic network and Australia all use this this model for their clinical islet transplant programs (17).

The minimum transplant requirement is ≥5000 islet equivalents (IEQ) per kg body weight of the recipient (9). Many islet preparations fulfill this criterion after isolation, but the functional islet mass often decreases below this threshold during culture and transport. Currently, it is estimated that 20–40% of functional insulin-producing islet tissue can be lost during the peri-transplant phase, where islets are cultured, quality tested and shipped to the transplant center (18, 19). It has been shown in several studies that islet transport and associated stress conditions such as high tissue density, mechanical force and oxygen deprivation can lead to islet damage and cell death, impacting on the functional islet mass for transplantation.

Improvement of islet survival during transport is therefore a critical step to increase the number of successful transplantations and to reduce the number of isolations required to enable each clinical transplant. Such an achievement would open this treatment to more patients and reduce the overall cost of the transplant procedure. An improved islet transport device could also be widely applied to emerging beta-cell replacement technologies, such as insulin-secreting tissues from adult and embryonic stem cells and islets from animal sources, which aim to alleviate organ shortage barriers.

Currently, cell culture bags made of gas permeable polymers such as poly(tetrafluoroethylene), poly(tetrafluoroethylene)co-(hexafluoropropylene) or silicone rubber membrane are employed for islet transport (20, 21). Freshly isolated islets are dispersed in a transport medium and loaded into the bags prior to transport to the transplantation centers (22). Conventional transport containers such as culture bags or flasks do not segregate or immobilize the islets; consequently, islets can freely move and interact with each other during transport. Such interactions induce aggregation and shear-fragmentation of the islets, thereby changing the shape, size and viability of the clusters. Larger islet aggregates have been shown to have a hypoxic core, potentially leading to the impairment of islet function and viability (23, 24, 25). Therefore, a transport device that will reduce islet aggregation and fragmentation is expected to improve the cell survival during shipping.

In this study, we report the development of a novel transport format that compartments islets on a poly(dimethylsiloxane) (PDMS) microwell array to reduce islet interactions. PDMS is an oxygen-permeable silicon rubber, allowing the high oxygen requirements of human islets to be supported (26). We show that the microwell device protected islets from aggregation during transport, maintaining viability and average islet size during shipping, whereas islets shipped under standard control conditions showed a loss in viability and average islet size.

## Methods and materials

### Microwell fabrication and characterization

#### Fabrication

The design of the microwell devices was created in Solidworks (Dassault Systems, France), a computer-aided drawing (CAD) software, then exported as a .STEP file for 3D printing of the casting molds. Casting molds were fabricated for a two-part device: a base, containing the microwell structures, and a lid used to enclose pancreatic islets during shipping studies. The molds were printed from temperature resistant HED525 plastic using a PolyJet printer (Objective 3D, Australia). Upon receipt, each part was thoroughly washed with deionized water and allowed to dry, then coated by plasma polymerization using the fluorosurfactant Zonyl (Sigma-Aldrich) (Supplementary Fig. 1, see section on supplementary data given at the end of this article). Plasma deposition was performed in a purpose-built capacitively coupled bell-chamber reactor (27, 28, 29). The cleaned 3D printed parts were added to the plasma reactor and the chamber brought to a vacuum. To further clean and prime the 3D printed parts, atmospheric air was introduced into the chamber until a steady pressure of 1.1/10 mbar was achieved. A plasma was ignited in air with a 50 W continuous wave (CW) radio frequency (RF) for 5 min. This process ensured that any unwanted organic material on the surface was removed prior to polymer deposition and activation. Subsequently, the pressure in the chamber was lowered to 100 mTorr and the Zonyl monomer was introduced via a needle valve until steady working flow rates were achieved. The plasma was ignited with a 20 W CW RF for 4 min. The Zonyl plasma polymer formed a non-adhesive layer on the mold surface, facilitating removal of the cast PDMS device. The plasma reactor and precise procedure have been previously reported (27, 28, 29). Because plasma polymer coating is substrate independent, a piece of silicon wafer was introduced into the chamber for each coating run facilitated, to enable subsequent characterization of the plasma coating using contact angle and ellipsometry. After plasma polymer coating, the device parts were left overnight at room temperature to allow the coating to settle. For PDMS casting of the device, Sylgard 184 (Corning) was mixed according to the supplier’s specification, and degassed under vacuum until a clear liquid was obtained. The Sylgard solution was then poured onto the coated molds and cured at 60°C overnight. The cured parts were removed from the mold and sonicated in acetone and ethanol for 20 min in each solvent. Finally, the parts were cured for 1 h at 100°C. A nylon mesh (Clear Edge Filtration, Tulsa, OK, USA) with a pore size of 50 µm used as the holding membrane was cut to size with a compass cuter. Prior to use, the parts were washed with 70°C ethanol and gamma irradiated (30 Gy) to ensure sterility.

#### Ellipsometry

Clean silicon wafers were exposed to the same plasma polymerization treatment as the 3D-printed mold parts and were used for further characterization of the plasma polymer coating. Coating thickness on the silicon wafers was determined via ellipsometry using a J. A. Woolam Co. (Lincoln, Nebraska, USA) variable angle spectroscopic ellipsometer (VASE). All measurements and data were analyzed using WVASE32 software provided with the instrument. Polymer thickness values were estimated by applying a Cauchy model (Supplementary Table 1).

#### Contact angle

A custom-built sessile drop apparatus with an Olympus SZ-PT microscope and lens system mated to a Sony CCD camera was employed to measure the wettability of the plasma-coated surfaces. A 10 µL syringe (Hamilton, Reno, USA) was used to dispense droplets of MilliQ water of approximately 1 µL on the blank or Zonyl plasma-coated silicon wafer. A minimum of three contact angle measurements were taken from each surface. Angle analysis of captured droplets was performed with ImageJ software, v1.50 with the DropSnake plugin (Supplementary Table 1).

#### Scanning electronic microscopy (SEM)

SEM images were obtained using a field emission SEM (Merlin, Zeiss, Germany), fitted with a GEMENI II column (Zeiss, Germany) and a secondary electron detector, operating at 1 kV in high vacuum mode. Measurements of microwell dimension were performed using the open source software ImageJ 1.50.

### Islet isolation and culture

#### Murine islets

Mouse islets of Langerhans were isolated from 6- to 12-week-old male or female C57Bl/6 mice using collagenase P (Roche) and Histopaque-1077 density gradients (Sigma-Aldrich) as previously described (30, 31). Islets were cultured at 37°C and 5% CO_2_ in RPMI (Sigma) or CMRL (Gibco-Invitrogen) supplemented with 2 mM l-glutamine, 100 U/mL penicillin, 100 U/mL streptomycin and 10% fetal calf serum. All animal experiments were approved by independent animal ethics committees of the University of Adelaide, SA Pathology and St Vincent’s Hospital, Melbourne.

#### Human islets

Human pancreata were obtained, with informed consent from next of kin, from heart-beating, brain-dead donors, with research approval from HREC committee at the St Vincent’s Hospital Melbourne. Human islets were purified by intraductal perfusion and digestion of the pancreas with collagenase followed by purification using Ficoll density gradients (32). Purified islets were cultured in Connaught Medical Research Laboratories (CMRL) 1066 medium (Invitrogen) supplemented with 10% human serum albumin, 100 U/mL penicillin, 100 mg/mL streptomycin and 2 mM l-glutamine (complete CMRL), in a 37°C, 5% CO_2_ humidified incubator.

### Shipping protocol and handling

After isolation, mouse islets were cultured on the PDMS microwell device in complete CMRL medium overnight at 37°C, 21% O_2_, 5% CO_2_. Control cultures, using standard non-adherent tissue culture plastic, were performed in parallel. The next morning, control islets were transferred into a 1.7 mL screw-cap microfuge tube. Tubes were filled with complete media before sealing. This is representative of shipping human islets in 50 mL Falcon tubes, a practice that is widely used in many isolating centers worldwide and an accepted reference shipping method (22, 33, 34). The PDMS devices were prepared for transport by inserting a permeable filter mesh to cover the islet-containing microwell surface, then filling the device with complete media before closing and sealing the device with the cast PDMS lid (Fig. 2). As shown in Supplementary Fig. 2A, the microwell device and microtube control were loaded into a stabilizing wire rack, which was then placed into a zip-lock plastic bag, closed to trap as much air as possible in the bag. This bag was then placed into a styrofoam box containing cold packs to stabilize temperature during shipment. Temperature fluctuation was monitored during shipping by a temperature probe (Supplementary Fig. 2B). Complete packages were shipped via a commercial courier service, involving road transport from the isolating center to the local airport and then a commercial flight to the destination city (730 km) and then road transport to the receiving center. These conditions exactly replicate our clinical islet transplant program (35). Upon arrival after transport or at the end of the pseudo-shipping period, the microwell device was transferred to a cell culture incubator at standard conditions (37°C, 21% O_2_, 5% CO_2_). Islets transported in the control device were transferred to a suspension 6-well plate and incubated in the same way overnight. For pseudo-shipping, conditions were simulated by 5–6 h on moving platform at a speed of approximately 60 rpm (denoted as pseudo-shipping conditions). To recover islets from shipping device, the mesh was removed and then gentle pipetting was used to allow islets to become suspended and collected. A final PBS wash of the wells ensured maximal collection.

### Islet size determination

After 24-h post-shipping islets were collected and bright-field images of the islets were obtained at 40× magnification (Nikon Eclipse Ti-U Microscope, DS-Qi2, Nikon). Using NIS-Elements BR Microsoft image software (Nikon), 100 islets were randomly selected from these images and measured. An average size for each shipped preparation was then calculated and used for further analysis.

### Human islet density and loading analysis

To quantify the efficiency of device loading, human islets were cultured on the microwells of different microwell size (300, 500 and 700 μm) and exposed to pseudo-shipping conditions (5–6 h on moving platform at a speed of approximately 60 rpm) at room temperature. The microwell devices were imaged before pseudo-shipping (pre-shipping) and after pseudo-shipping (post-shipping) on an upright microscope (Olympus CKX41). The number of islets per microwell was manually counted for each microwell array design over six fields of views and the distribution of islet content per microwell was then calculated. For the other assays, the islets were removed from the shipping devices to carry out the assays according to state-of-the-art protocols.

### FDA/PI viability assay

Mouse islet samples were stained to determine whole islet viability with fluorescein diacetate (FDA, Sigma-Aldrich) and propidium iodide (PI, Sigma-Aldrich). In brief, islets were transferred to a 24-well suspension plate in 400 µL volume, PI and FDA were added to achieve a final concentration of 5 µg/mL and 5 µM, respectively. Samples were incubated at room temperature for 5–10 min and imaged as soon as possible. Fluorescent images were taken using the Nikon Eclipse Ti-U Microscope (DS-Qi2) (Nikon) and the NIS-Elements BR Microsoft image software (Nikon) was used for image analysis.

### Oxygen consumption rate

Oxygen consumption rate (OCR) was determined using a commercially available Micro Oxygen Uptake System (Instech Laboratories, Plymouth Meeting, PA, USA), a method described in detail by Papas and colleagues (36). Briefly, OCR of murine islets was determined from the linear decrease of pO_2_ over time, which was then normalized to DNA content of the islets in each sample using the Quant-iT PicoGreen dsDNA Assay kit as per manufacturer’s instructions (Invitrogen, Life Technologies).

### Quantitative real-time PCR

Extraction and purification of RNA was performed using RNAqueous Micro Kit (Ambion cat no AM1931) according to the manufacturer’s instructions. The purified RNA was quantified by absorbance at 260 nm using a NanoDrop 2000. RNA (200–500 µg) was reverse transcribed in a 20 µL reaction using iScript Reverse Transcription Supermix for RT-qPCR (Biorad Cat no. 1708841) according to the manufacturer’s instructions. The complete reaction mix was diluted 1:2 following an incubation of 5 min at 25°C, 30 min at 42°C and 5 min at 85°C. Real-time quantitative PCR analysis was performed on duplicate or triplicate samples, using 2 µL of cDNA using the following gene-specific TaqMan primers (Applied Biosystems; Life Technologies): *Mcp-1* (*Ccl2* (Mm00441242_m1)), *Glut-2* (Slc2a2 (Mm01333430_m1; Mm00446229_m1)), *Ldha* (Mm1612132_g1), *Il6* (Mm00446190_m1), *Pdx1* (Mm00435565_m1) and *Insulin* (Mm01950294_s1). *B-actin* (ACTB Mm02619580_g1) was used as a housekeeping gene and to normalize expression data using 2^−Δct^ as a method of quantitation.

### Statistical analysis

Data were analyzed using Prism 6 (Graph pad). Results are expressed as mean ± s.e.m. or ±s.d. as indicated by individual figures legends. A value of *P* < 0.05 was considered statistically significant and all *P* values reported were two-sided, unpaired Student’s *t*-test or as indicated in the figure legend.

## Results and discussion

**Figure 1:**
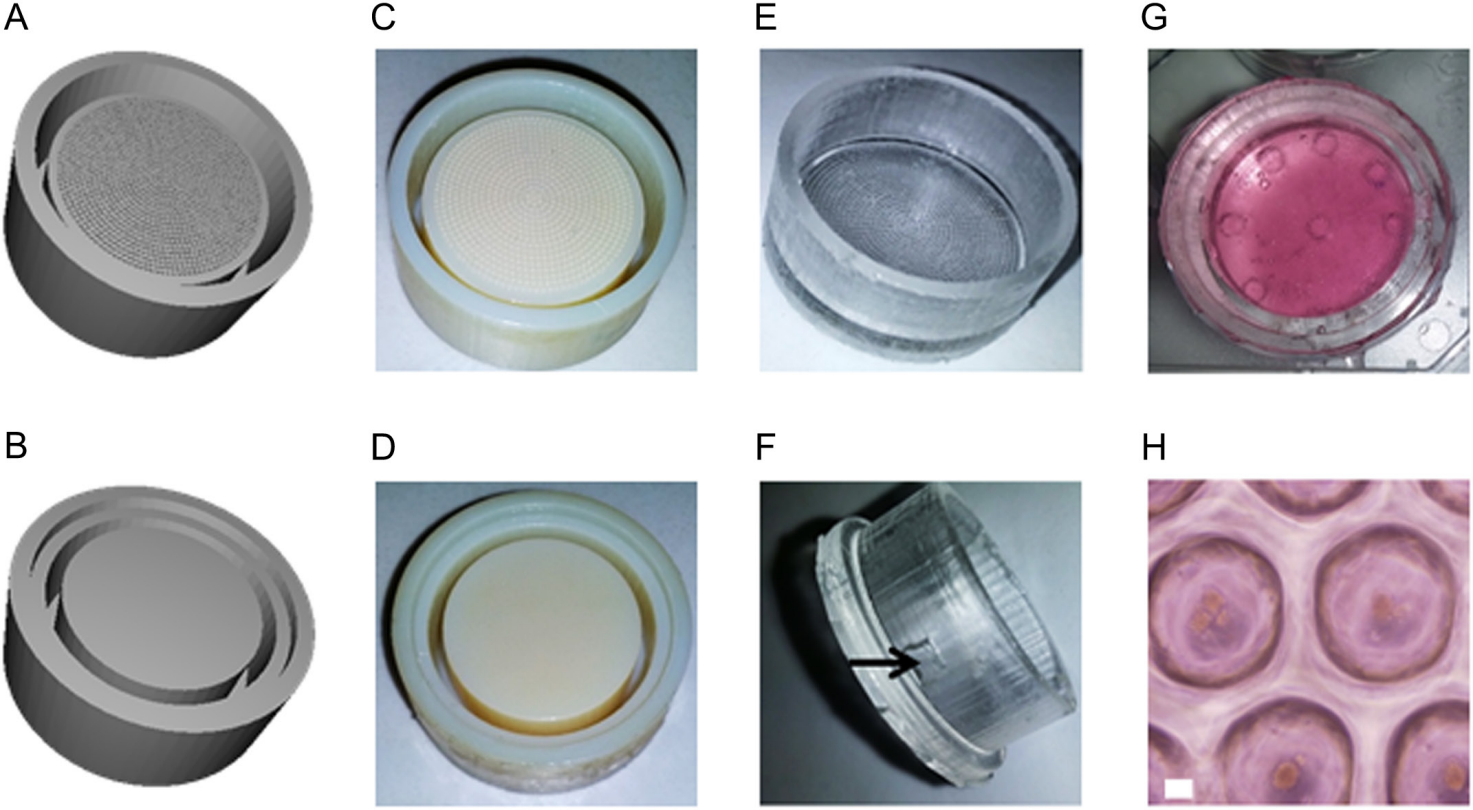
3-D rendering of the computer-aided drawings of (A) the microwell mold and (B) the lid mold. Photograph of (C) the 3-D printed microwell mold and (D) the lid mold. Polydimethylsiloxane (PDMS) (E) casted microwell inserts and (F) lids closing the device base, the arrow shows the side opening for complete loading of the device. The PDMS molds fit into 6-well plate format, modified with opening at the top and bottom to allow gas exchange. (G) Photograph of the loaded PDMS microwell device. (H) Representative photograph of islets within the microwells.

Most published microwell arrays have been made from polymeric materials, including collagen (37), agarose (38, 39), poly(ethylene glycol) (PEG) (40), poly(butylene terephthalate)-co-(ethylene oxide terephthalate) (PEOT/PBT) (41) or PDMS (39, 42). While the biocompatibility of these materials is comparable, PDMS allows greater oxygen diffusion and the platform described here could be further developed to incorporate oxygen releasing coating as previously reported for PDMS (43). Thus, PDMS was the material of choice in this study due to its high oxygen permeability and facile fabrication (39, 44). With CAD software, we designed a mold for a two-part container with a base and a lid (Fig. 1A and B). To allow for incorporation within conventional laboratory tissue culture plastic, the container can be fitted into a 6-well plate. The microwells were imprinted in the base of the device. The device lid was constructed to hold a semi-permeable membrane in close contact with the imprinted base, in order to restrict movement of the islets from one well to another during shipping but allowing for exchange of nutrients and oxygen, offering a considerable advantage compare to current transport protocols where islets can freely move in a culture bag or a flask. The molds were 3D-printed from urethane-acrylate, a heat-resistant material, to be compatible with PDMS curing at 60°C (Fig. 1C and D). However, after curing, PDMS became adherent to the 3D printed mold. Therefore, the mold was coated with a fluorinated plasma polymer using perfluoro-1-hexene (Zonyl) as monomer. This coating facilitated the release of the PDMS parts out of the mold. This technique allowed us to quickly obtain the PDMS parts utilized for the transport device: bottom microwells and lid (Fig. 1E and F). The transport device could then be loaded with the islet suspension and closed with the lid (Fig. 1G and H). Because the PDMS is a polymer network, the microwell base and the lid formed a watertight seal on contact.

**Figure 2:**
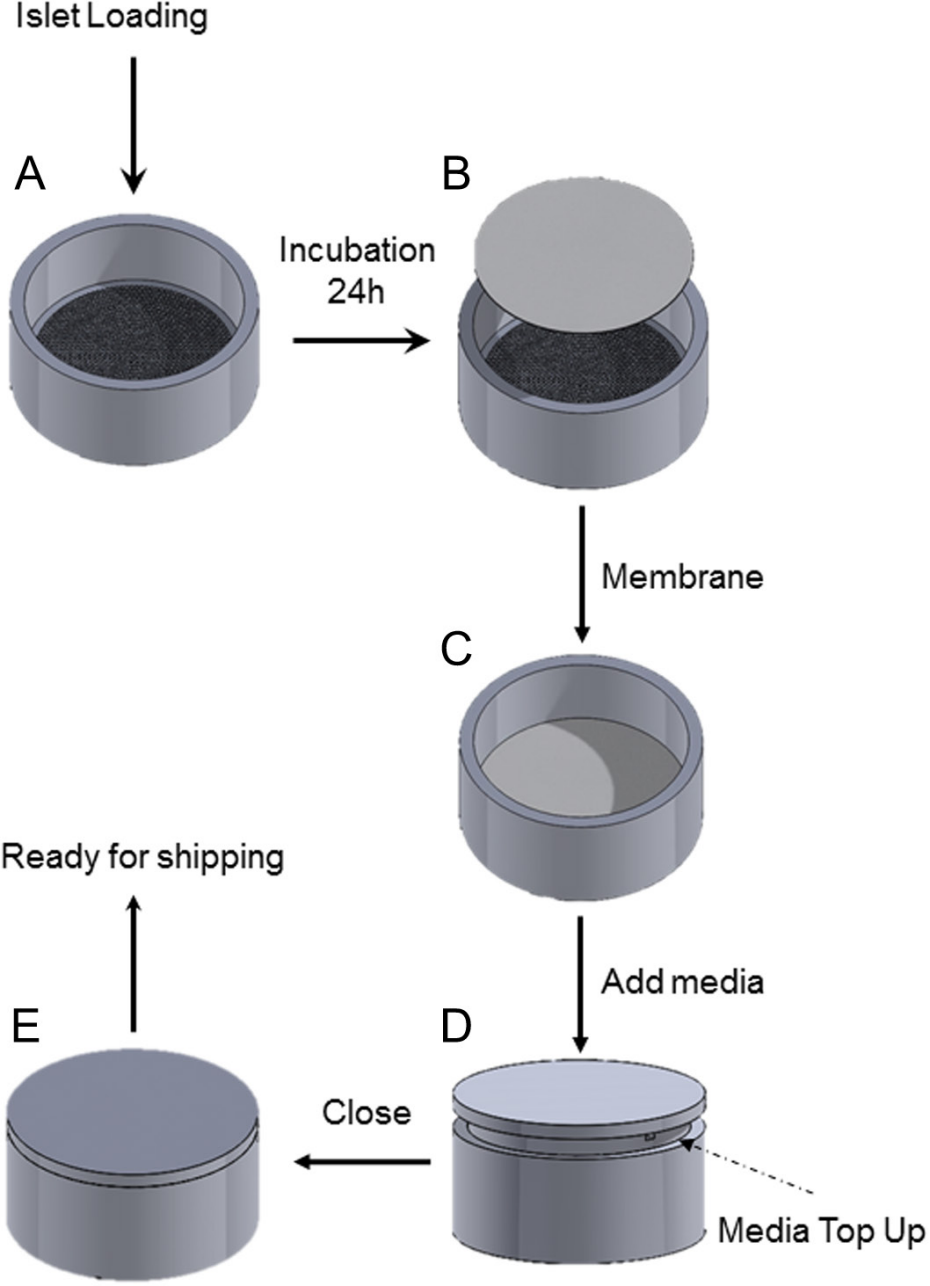
3-D rendering of the computer-aided drawings representing the islet loading process within the microwell array. (A) The islet suspension was loaded on a microwell array that is then incubated for 24 h at 37°C, (B) then a 50 µm membrane was deposited on top of the microwells and (C) extra transport medium was added to top up as much as possible. (D) Then, the lid was placed on the top of the microwell array leaving the side opening free to allow for media top up. (E) Finally, the lid was gently closed forming a watertight seal and the device was placed with a 6-well plate, ready for shipping.

Subsequently, we developed a protocol for the efficient loading of the transport device with islets (Fig. 2). The PDMS microwells were loaded with an islet suspension and the islets were allowed to settle into the microwells overnight. The islets do not adhere to the PDMS; thus, the islets can freely move out of the microwells. To limit movement of the islets out of the microwells, a semi-permeable membrane with a 50 µm mesh was positioned on top of the microwells. The device was loaded with cell culture media and the lid was placed inside the device. By tilting the device, the device could be completely filled with culture media, and air evacuated. When fully inserted, the lid not only closed the device but also held the membrane in position on top of the microwells. This last manipulation was critical, as the presence of an air bubble can expose the islets to air and limit access to nutrients if the device is tilted during the transport. Successful loading of the islets in the microwell device was assessed by observation using an upright microscope (Fig. 1G and H).

Given the clear consensus that anoxic conditions are detrimental to the overall health and function of islets (24), gas permeable bags are often employed for islet culture and transportation pre-transplant (22). However, islets transported in semi-permeable bags showed significant loss of islet integrity, despite having comparable viability to a non-permeable blood transfusion bag. It was postulated that the shear stress from the bag movement during transport leads to the change in islet integrity (45). Moreover, these bags are difficult to handle, promote the aggregation of islets and have a propensity to rupture and become contaminated.

To avoid islet aggregation and fragmentation, we created a closed device housing an array of microwells, sized to spatially isolate islets during transport. Implantable microwell scaffold arrays have previously been reported to improve cell survival for extrahepatic implantation of islets (41). In addition to being tested as an implantable platform, such microwell arrays have also been extensively used in the formation of β-cell aggregates from dissociated pancreatic islets (40, 46, 47) or from the insulin-producing MIN6 cell line (48, 49, 50). These microwell platforms, however, were fabricated using expensive manufacturing techniques such as soft lithography or hot embossing, which do not allow for rapid prototype generation. Here, we generated a prototype microwell shipping device using inexpensive techniques with a tunable microwell diameter.

**Figure 3:**
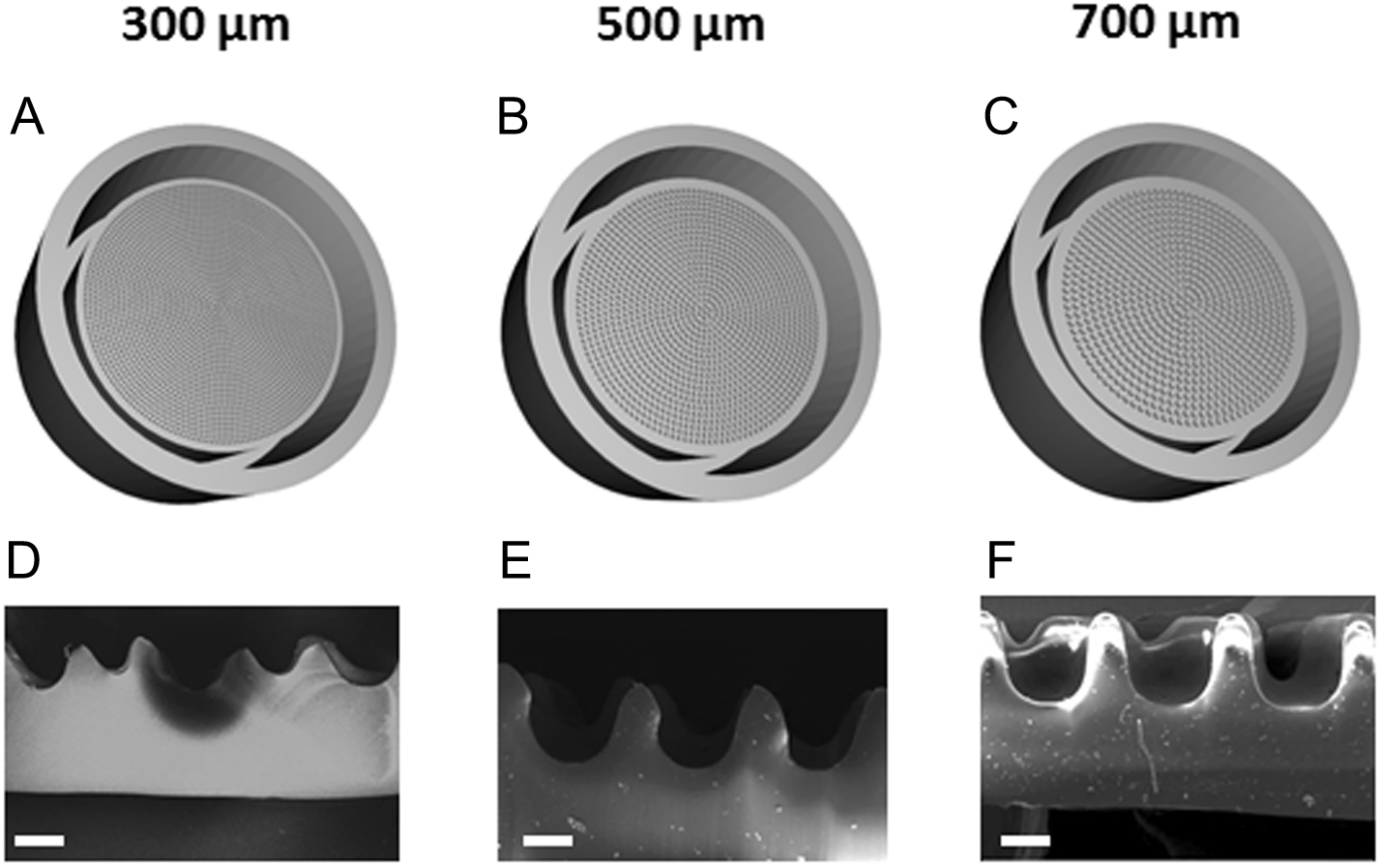
3-D rendering of the CAD of the mold used to make the microwells array of different sizes with microwells sizes of 300, 500 and 700 µm (A, B and C). Scanning electron microscope (SEM) micrographs of the microwell cross-section (D, E and F) 300, 500 and 700 µm, respectively. Scale bar 250 µm.

Theoretically, optimal islet segregation during transport is achieved when each microwell houses a single islet. However, due to the size distribution of human islets, ranging from 50 µm to over 350 µm (51) and in some instances up to 600 µm, it was expected that microwells may harbor several islets. In order to determine the optimal microwell diameter configuration – minimizing clustering of small islets while being large enough to accommodate larger islets – we designed three different microwell array designs with well diameters of 300, 500 and 700 µm (Fig. 3A, B and C). The microwell cross-section was imaged by SEM to verify the successful imprinting of the mold (Fig. 3D, E and F). In each case, the depth of the microwells was identical: 500 µm.

**Figure 4:**
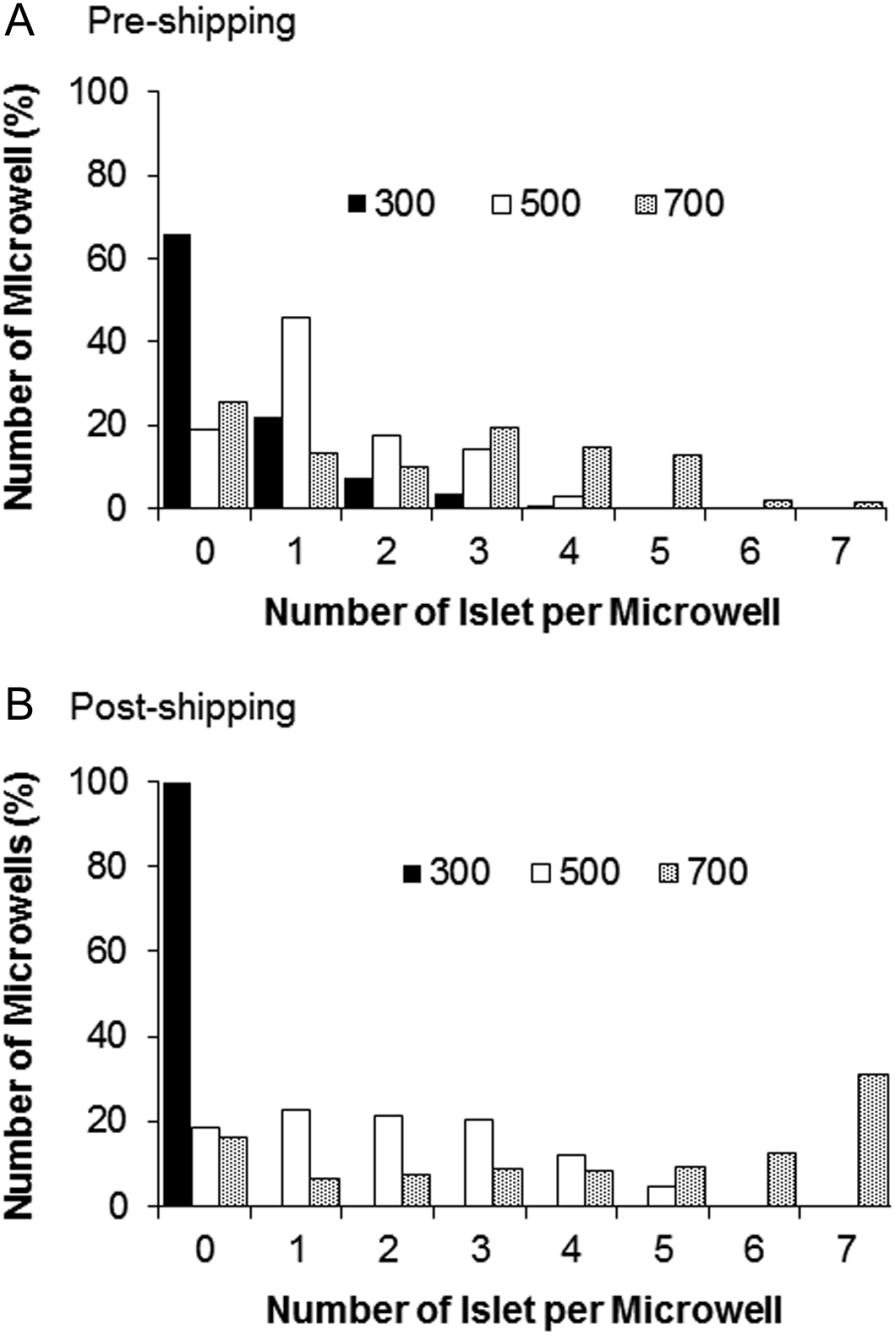
Islet distribution within 300, 500 and 700 µm microwell arrays (A) before pseudo-shipping experiment and (B) after pseudo-shipping experiment. The microwell device is able to maintain islet size and hypoxia-related gene expression at basal levels during simulated shipping.

Human islets were used to assess islet loading and distribution over the different microwell sizes under simulated shipping conditions. As described in detail in the Materials and Methods section, the number of islets contained per well was counted for each microwell size, pre-simulated and post-simulated shipping (Fig. 4 and Supplementary Table 2). For the smallest microwell diameter assessed (300 µm), few islets entered the microwells upon loading, and these were not retained within the microwells during pseudo-shipping. The majority of islets aggregated on the surface of the device and did not enter the microwells. In contrast, microwells of 700 µm diameter housed multiple islets per well (around 4 islets per microwell on average) after loading and pseudo-shipping. The capacity of the 500 µm diameter microwells was sufficient to load islets efficiently, while segregating islets into individual wells (1.4 and 1.8 islets per microwell before and after simulated shipping, respectively, (Supplementary Table 2). As the 500 µm microwell format produced optimal human islet loading and segregation, we chose this format for further investigations of islet viability and function.

**Figure 5:**
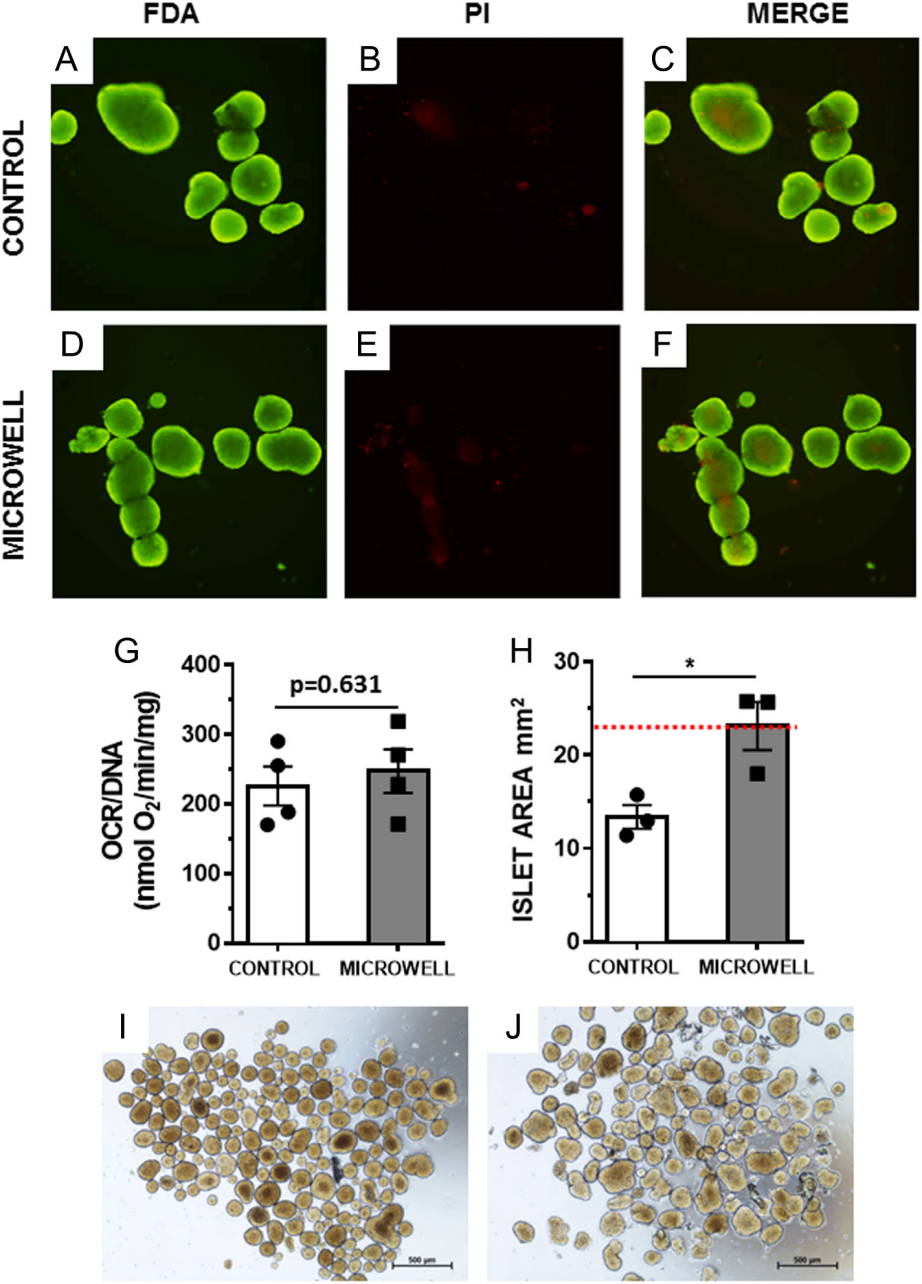
Shipping of murine islets under pseudo-shipping conditions. Islets were subjected to pseudo-shipping, and then cultured for 24 h and assessed for whole islet viability using fluorescent FDA and PI, for islets from the control tube (control) (A and B) and islet shipped in the microwell device (D and E), and overlays (C and F), representative of 3 independent experiments, 100× magnification. Mean OCR of islets subjected to pseudo-shipping in control tube or on microwell device is shown in (G) from 4 independent experiments +/− s.e.m. Mean area per islet was determined by randomly selecting 100 islets per preparation, data from three independent experiments is represented in graph (H), *P < 0.05, two-tailed *T*-test, error bars +/− s.e.m., where the red-dash line is representative of the average size of unshipped islets. Bright-field images of the islets after removal from microwell device (J) and control tube (I).

To provide standardized islet tissue for assessment of the shipping device, we used islets from C57Bl/6 mice to assess the biological responses following shipment in our prototype device. Islet viability and function were compared after shipment of islets on the microwell devices and under standard reference conditions in non-gas-permeable tubes (22, 33, 34). We determined islet viability of murine islets subjected to pseudo-shipping for 5 h, a time period that reflects the transportation time of islets between isolation and transplanting centers using commercial carriers based on our experience (Supplementary Table 3) and others (52). Islets were collected and analyzed directly after pseudo-shipping and also after 24-h culture post pseudo-shipping. As shown in Fig. 5, assessment of islet viability with FDA/PI did not reveal a significant difference between islets from the control tube (Fig. 5A, B and C) and islets from the microwell device (Fig. 3D, E and F). Despite being an accepted viability test for clinical preparations, detection of subtle viability changes using FDA/PI has been a common challenge in the field of islet biology (53, 54). It has been shown that the OCR of islets can reveal variability in preparations that otherwise cannot be separated by FDA/PI (55). This method has recently been proposed as a predictive marker to determine islet quality prior to transplantation (56, 57). For this reason, we also used OCR/DNA ratio as a measure of metabolic rate and a surrogate marker of islet viability. No difference was observed in the OCR of islets 24 h after pseudo-shipping (Fig. 5G), suggesting a similar oxygen metabolic rate in islets from both the device and control tubes following simulated transport.

We performed size analysis of 100 randomly selected islets per image of islets from three independent experiments after pseudo-shipping in the microwell device and control tubes. As shown in Fig. 5H, islets shipped in microwell devices were significantly larger on average than islets from control tubes (*P* = 0.027). Given that for each experiment, islets shipped by each method originated from pooled starting populations of isolated murine islets, this indicates that islets from control tubes have a reduced islet mass. Islets shipped within the microwell device retained a similar size to islets that did not undergo islet shipping (indicated by the red-dash line, Fig. 5H). Bright-field images of islets from microwell device (Fig. 5J) and control tube (Fig. 5I) revealed subtle differences in islet morphology. Islets incubated in control tubes exhibited ruffled edges compared to islets from microwell devices.

**Figure 6:**
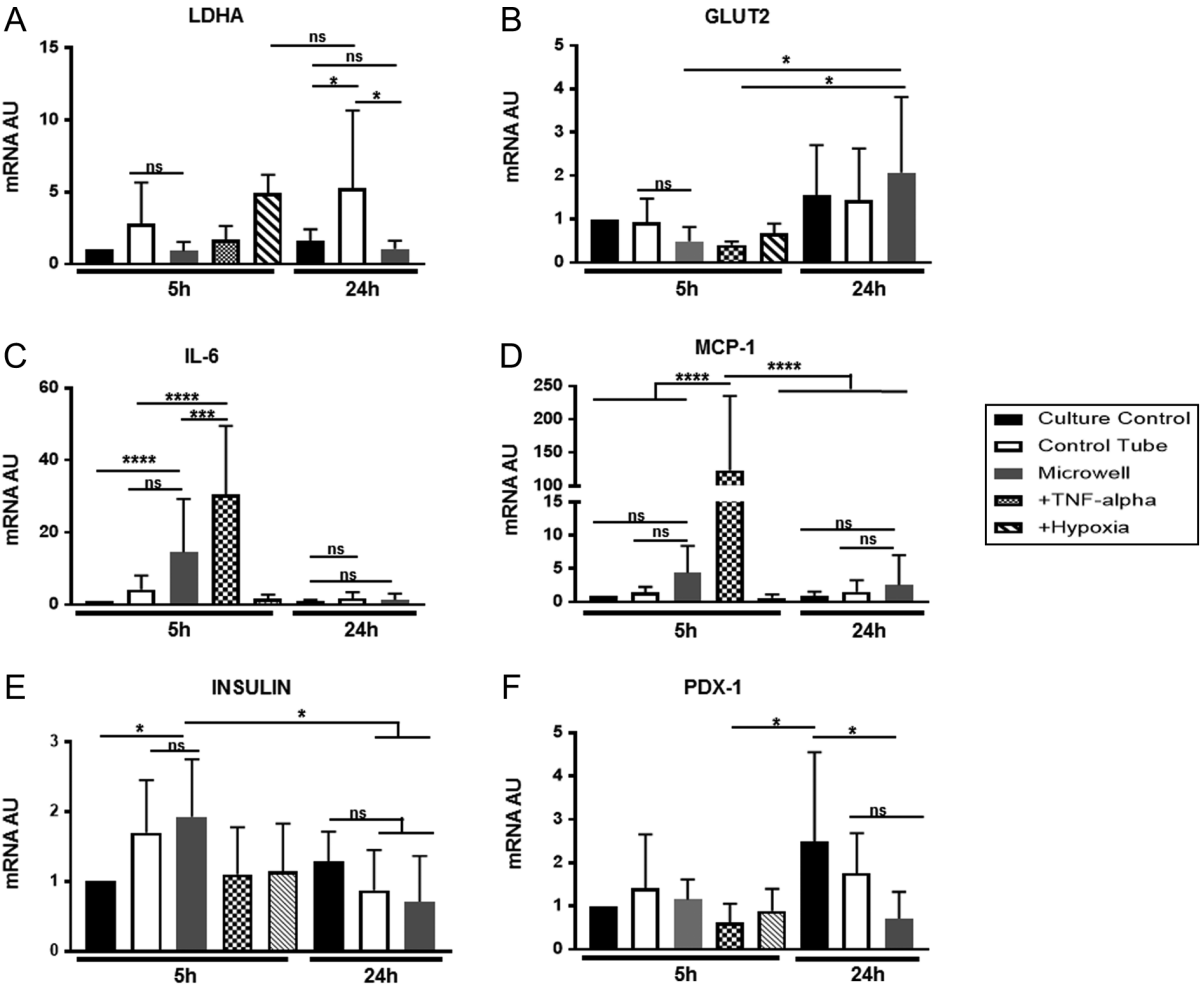
Islet mRNA gene expression after pseudo-shipping. Islets were collected either directly after the 5-h pseudo-shipping period (white bars, *n* = 4 independent experiments) or after a 24-h culture period (grey bars, *n* = 6 independent experiments). Islets cultured under standard culture conditions (culture control, black bars), exposed to 2% O_2_ (+hypoxia, *n* = 3, diagonal lines) or 100 ng/mL of TNF-alpha (TNF-alpha, *n* = 3, checkered) for 5 h were used as controls. RNA expression was analyzed by qRT-PCR for Ldha (A), Glut2 (Slc2a2) (B), Il6 (C), MCP1 (Ccl2) (D), insulin (Ins) (E) and Pdx1 (F), normalized to the housekeeping gene beta-actin. Results are shown as mean + s.d. of mRNA fold change from 5 h culture control. Statistics: One-way ANOVA, multiple comparisons, uncorrected Fisher’s LSD test where *P ≤ 0.05; ***P ≤ 0.001; ****P ≤ 0.0001.

It has been shown that the transcriptional profile of human islets during isolation and culture reflects changes in viability and function in response to two major stress factors, inflammation and hypoxia (56, 58, 59, 60, 61). These transcriptional changes are conserved in model species (rat and mouse) (62, 63, 64). We analyzed validated markers of islet viability (*Ldha*, *Slc2a2*), inflammatory response (*Ccl2*, *Il6*) and islet differentiation/function (insulin (*Ins*), *Pdx1*) in murine islets subjected to pseudo-shipping. Samples were collected directly at the end of the 5-h pseudo-shipping period, and again at 24 h post shipping. Islets cultured under standard culture conditions, exposed to hypoxia (2% O_2_, 5 h) or the cytotoxic cytokine TNF-alpha (5 h) were used as controls for induction or loss of target gene RNA expression, respectively. Expression of lactate dehydrogenase A (*Ldha*) is suppressed in mature beta-cells and associated with anaerobic metabolism (45, 58, 65). Islets pseudo-shipped in control tubes or microwell device showed no significant changes to *Ldha* transcription in relation to culture control after 5 h of pseudo-shipping (Fig. 6A).

However, after 24-h *Ldha* transcription levels significantly increased in islets from control tubes compared to the 24-h culture control and the islets shipped in the microwell device (*P* = 0.0218 and *P* = 0.0133 respectively). This level of *Ldha* transcription in islets from control tubes was comparable to the positive hypoxia control (*P* = 0.870). The expression of glucose transporter 2 (*Slc2a2*) an essential glucose-sensing molecule (66, 67) was also investigated. Directly after pseudo-shipping, there were no significant changes in Glut2 gene expression between the culture control and the test groups. However, Glut2 expression of islets shipped in the microwell device did significantly increase at 24 h when compared to the sample taken at 5 h. No significant changes were observed at 24 h between the groups (Fig. 6B). Expression of the cytokine *Il6* (Fig. 6C) was transiently induced after shipping in microwells after 5 h when compared to culture controls, but not the control tube. This slight rise in IL6 transcript levels then returned to basal levels after the 24-h culture period. Similarly, there was a slight rise in expression of the chemokine Mcp1 (*Ccl2*) in islets from control tubes (Fig. 6D) at 5 h, which then decreased at 24 h, though changes were not statistically significant. Analysis of the mRNA expression of insulin (*Ins*) (Fig. 6E) showed no significant changes in islets pseudo-shipped in control tubes compared to that on the microwell device. There was a slight but significant increase in insulin mRNA in the microwell-shipped islets when compared to culture control islets. However, this trend did not persist at 24 h, where the expression was similar in all groups. Islet differentiation transcription factor *Pdx1* showed a small significant decrease at 24 h in microwell shipped islets when compared to the culture control. However, this was not statistically different to islets from the control tube (Fig. 6F).

Overall, our results indicated that the microwell device retained high viability in healthy mouse islets under simulated shipping conditions. Induction of stress responses was moderate and transient. We next tested the capacity of the microwell device to support islet integrity and survival under real shipping conditions, reflecting the process currently used for human islet transplantation.

**Figure 7:**
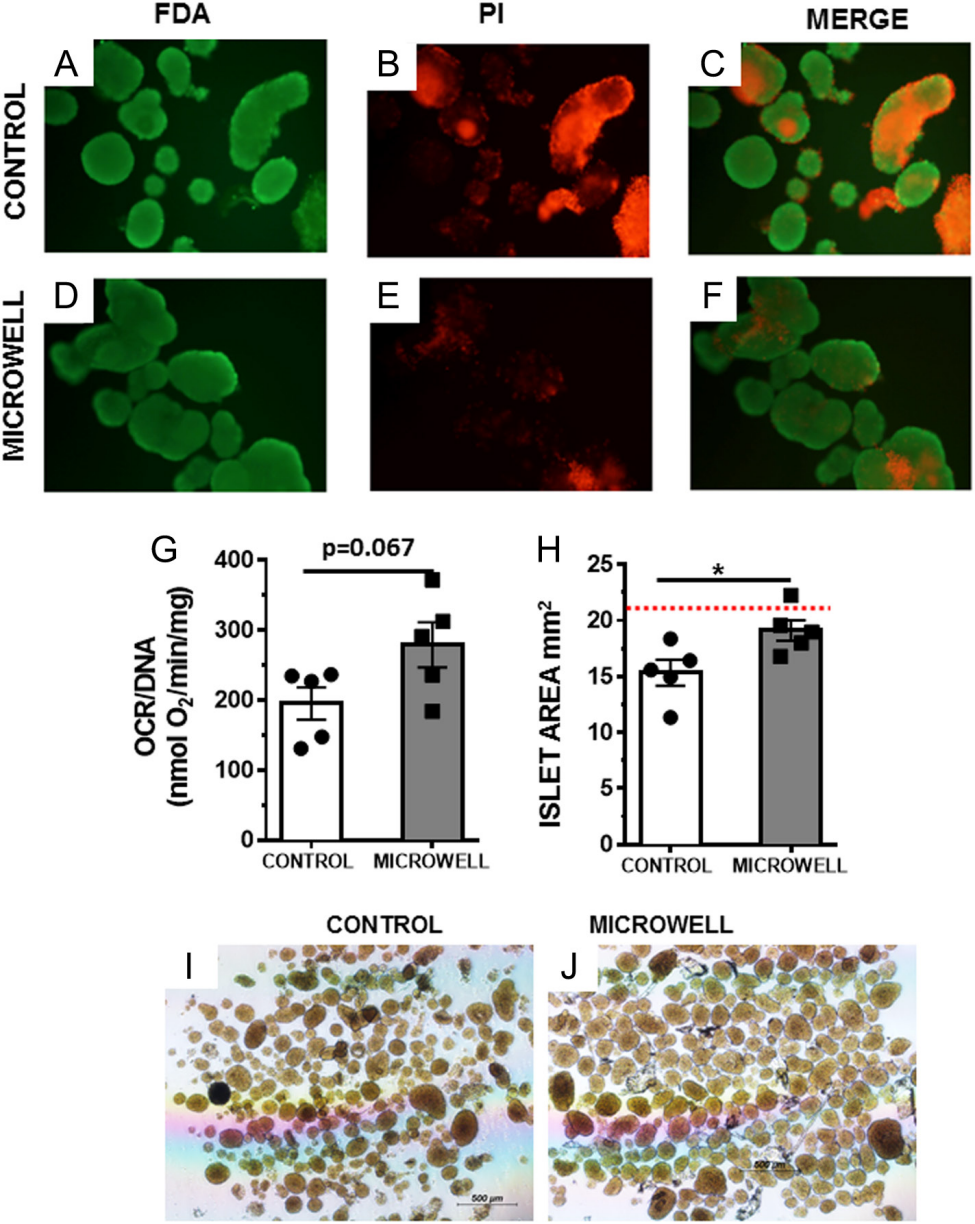
Long-distance shipping of murine islets. Whole islet viability with FDA and PI was determined after shipping and an overnight culture period. Images shown include separated channels and overlay, control tube (control) (A, B and C) and islet shipped in microwell device (D, E and F), 100× magnification. FDA/PI images are representative of 3 independent shipping experiments. Oxygen consumption rate normalized to DNA of the islets transported in the control tube and in the microwell device (G), and islet area measured from 100 islets selected randomly per condition (H). OCR and islet area imaging were performed 24 h post-shipping arrival at receiving center. (H) and (I) Show representative bright-field images taken at 40× magnification. (G) and (H) Show data from 5 independent experiments mean+/− s.e.m., statistical analysis was completed with GraphPad Prism software. *P < 0.05, two-tailed *T*-test.

To demonstrate the performance of the microwell device under real-world long-distance shipping conditions via air and road, we transported mouse islets in the microwell device over a distance of approximately 750 km (460 miles), with an average of 5.36 ± 0.51 h in transit (Supplementary Table 3) using a commercial courier. Islets were packaged according to the clinical guidelines for transportation (as described in the Materials and Methods section). Islets were shipped under temperature controlled conditions where shipments experienced stable temperature conditions (10–15°C, Supplementary Fig. 2B) throughout the transportation period. For comparison, islets were shipped in control tubes as part of the same consignment. Islets shipped in the microwell device had less PI-positive cell staining (Fig. 7D, E and F) compared to those control shipped (Fig. 7A, B and C), indicating better survival post transport. We also observed that islets shipped in the microwell device showed a trend toward improved OCRs (Fig. 7G) compared to tube control-shipped islets. Although this difference failed to reach significance (*P* = 0.067), four of five independent experiments showed increased OCR values for islets shipped in the microwell device, with improvements ranging from approximately 19–58% compared to controls (Supplementary Fig. 2C). Moreover, when assessing islet mass, we found that microwell device shipped islets also retained a larger size during shipment (Fig. 7H). The average size of islets shipped in the microwell device was not significantly different from those cultured in normal conditions for the duration of the experiment.

These data suggest that the 500 µm microwell size is optimal for loading of human islets while preventing islet crowding in the wells. Additionally, the semi-permeable membrane positioned on top of the microwells restricted the movement of islets between wells. We found that murine islets transported in the microwell device indeed showed improved viability according to FDA/PI staining and also a clear trend for improved OCR/DNA ratio, where 4 out of 5 shipments showed an improvement. Under both simulated and actual shipping conditions, the device demonstrated a significant maintenance of islet size, indicating that the physical separation provided by the microwell device has a positive effect on islet integrity. Currently, up to 40% of the islet mass can be lost during culture and transport and result in a preparation becoming unusable for infusion into the recipient (18, 19). Therefore, maintenance of islet viability and integrity during the peri-transplant period is vital to ensure that a preparation is transplantable and is able to achieve the most favorable clinical outcome.

In conclusion, we have successfully generated a microwell device that supports the effective transport of islets, where segregation provided by the microwells prevented aggregation and fragmentation. We believe that the lack of aggregation observed in our microwell device helped maintain the expression of *Ldha* at basal levels in islets transported in the microwell device. More importantly, we have shown that improved transport conditions facilitated by our device can effectively limit the loss of islet mass and therefore potentially maintain the number of transplantable islets. The improvement of islet mass retention is critical in making this treatment more widely available, as currently some patients require up to six islet infusions to become insulin independent (68). Nonetheless, as the field moves from cadaver organs to other forms of stem cell and animal-derived insulin-producing cell sources, this device has the capacity to provide superior transport conditions compared to the current state-of-the-art devices, in a relatively cost-effective format.

## Supplementary Material

Supporting Figure 1

Supporting Figure 2

Supporting Table 1

Supporting Table 2

Supporting Table 3

## Declaration of interest

The authors declare that there is no conflict of interest that could be perceived as prejudicing the impartiality of the research reported.

## Funding

This work was funded by the Cooperative Research Centre for Cell Therapy Manufacturing.
