# Supplementary material for: Oxygen-permeable microwell device maintains islet mass and integrity during shipping
Source: Endocr Connect. 2018 Feb 26;7(3):490–503. doi: 10.1530/EC-17-0349 (PMC5861371; doi:10.1530/EC-17-0349)
Supplement: Supporting Figure 1 [file ec-7-490-s001.pdf]

Fig. S1

A B

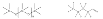

Fig. S1: Chemical structure of (A) poly(dimethoxysilane) polymer used to fabricate the transport device and (B) Zonyl® monomer used for the polymer plasma coating of the 3D printed mold.
