# Supplementary material for: Oxygen-permeable microwell device maintains islet mass and integrity during shipping
Source: Endocr Connect. 2018 Feb 26;7(3):490–503. doi: 10.1530/EC-17-0349 (PMC5861371; doi:10.1530/EC-17-0349)
Supplement: Supporting Figure 2 [file ec-7-490-s002.pdf]

Fig. S2

A

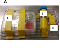

B

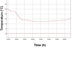

C

Shipment (n=5)	Control	Microwell
1	130.9934	312.5721
2	147.4802	184.0048
3	226.6749	371.7047
4	236.49	290.83
5	234.4737	235.9242

Fig. S2: Interstate shipping conditions of mouse islets. A) Image depicting the set up of the microwell device for shipping, highlights the 3 main components : 1- microwell device 2- temperature probe 3- Control device (1.5ml Eppendorf). B) Temperature probe measurements over the shipping time, representative graph from 5 shipments. C) OCR measurements from individual experiments/ shipments, measure expressed as OCR/DNA (nmol O2/min/mg).
