# Supplementary material for: Oxygen-permeable microwell device maintains islet mass and integrity during shipping
Source: Endocr Connect. 2018 Feb 26;7(3):490–503. doi: 10.1530/EC-17-0349 (PMC5861371; doi:10.1530/EC-17-0349)
Supplement: Supporting Table 1 [file ec-7-490-t001.pdf]

Table. S1: Contact angle measurements. Contact angle measured on the silicon wafer (blank) and Zonyl® plasma polymer coating for different deposition time on a silicon wafer showing an increase of contact angle and coating thickness measured by ellipsometry with increase of deposition time.

**Conditions**	**Time**	**Power**	**Vacuum**	**Thickness**	**Contact Angle**	
Blank	_	_	_	_	74.68˚	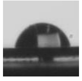
Short	2 min	20 W	100 mTorr	25 nm	86.90˚	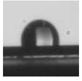
Long	4 min	20W	100 mTorr	80 nm	91.98˚	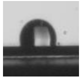
