# Supplementary material for: Oxygen-permeable microwell device maintains islet mass and integrity during shipping
Source: Endocr Connect. 2018 Feb 26;7(3):490–503. doi: 10.1530/EC-17-0349 (PMC5861371; doi:10.1530/EC-17-0349)
Supplement: Supporting Table 2 [file ec-7-490-t002.pdf]

Table. S2: Average islet per microwells on the different array format before and after simulated shipping.

Microwell size (µm)	*Pre-simulated-shipping*<br>*Average Islets per well*	*Post simulated shipping*<br>*Average Islets per well*
300	0.51	0
500	1.37	1.75
700	3.21	4.19
