# Supplementary material for: Oxygen-permeable microwell device maintains islet mass and integrity during shipping
Source: Endocr Connect. 2018 Feb 26;7(3):490–503. doi: 10.1530/EC-17-0349 (PMC5861371; doi:10.1530/EC-17-0349)
Supplement: Supporting Table 3 [file ec-7-490-t003.pdf]

Table. S3: Average transportation times. Calculation of average time taken for shipment of islets to travel from Melbourne to Adelaide using a commercial courier. Packages travel via air and road under temperature controlled conditions.

**Shipment (n=9)**	**Pick-up Time**	**Arrival Time**	**Hours**
1	09:00	15:30	06:30
2	10:50	17:40	06:50
3	09:15	14:55	05:40
4	09:15	14:15	05:00
5	10:00	14:20	04:20
6	09:30	14:25	04:55
7	10:30	16:40	06:10
8	08:00	14:05	06:05
9	11:15	16:10	04:55
		**Average**	**05:36**
		**SD**	**00:51**
